# Morphological and histological identification of *Paramphistomum cervi* (Trematoda: Paramiphistoma) in the rumen of infected sheep

**DOI:** 10.14202/vetworld.2015.125-129

**Published:** 2015-01-30

**Authors:** Vijayata Chaoudhary, J. J. Hasnani, Mukesh K. Khyalia, Sunanda Pandey, Vandip D. Chauhan, Suchit S. Pandya, P. V. Patel

**Affiliations:** 1 Department of Veterinary Parasitology, College of Veterinary Science & Animal Husbandry, Anand Agricultural University, Anand - 388 001, Gujarat, India; 2 Department of Veterinary Pathology, College of Veterinary Science & Animal Husbandry, Anand Agricultural University, Anand - 388 001, Gujarat, India

**Keywords:** abattoirs, histology, morphology, paramphistomosis, posterior sucker, rumen fluke, sheep

## Abstract

**Aim:**

This study was undertaken to identify *Paramphistomum cervi* on the basis of its morphology and histology to be the common cause of paramphistomosis in infected sheep and its differentiation from other similar Paramphistomes in Gujarat.

**Materials and Methods:**

Adult rumen flukes were recovered from the rumen of naturally infected sheep slaughtered in various abattoirs in Gujarat. Some adult flukes were flattened and stained in Borax carmine, and some were sectioned in the median sagittal plane and histological slides of the flukes were prepared for detailed morphological and histological studies.

**Result:**

Microscopic pictures of the parasite used in identification define the similarity in the morphology and histology of the anterior sucker, pharynx, esophagus, genital atrium, posterior sucker (acetabulum) and testes to the *P. cervi*.

**Conclusion:**

It can be concluded that the most common species found in sheep infected with Paramphistomosis is P. cervi on the basis of its histo-morphological appearance in Gujarat.

## Introduction

*Paramphistomum* spp. are Platyhelminth (flatworm) parasites (Platyhelminthes: Trematoda: Digenea) responsible for Paramphistomosis i.e. gastrointestinal parasitic disease in domesticated animals, which causes heavy economic losses [1] to the livestock industry to the tune of several thousand crores of rupees annually [2]. It has been a neglected trematode infectious disease in ruminants, but has recently come out as a significant cause of productivity loss [3-7]. Distribution of Paramphistomosis is worldwide [8], but the highest prevalence has been accounted in tropical and subtropical regions, particularly in Africa, Asia, Australia, and Eastern Europe [1,9].

It is caused by specific species of the parasite depending on the regions [3]. The common rumen fluke, *Paramphistomum cervi* is considered to be one of the most important species of Paramphistomes [2]. Adult flukes are located in the rumen of ruminants and immature flukes in the small intestine mainly in the duodenum [1,10]. *P. cervi* has di-heteroxenous life cycle, which involve snail (*Bulinus* spp., *Glyptanisus gilberti, Indoplanorbis exustus, Planorbis planorbis* and *Lymnaea bulimoides*) as intermediate host and mammalian as definitive host [11,12].

*P. cervi* are plug feeders [9] and cause serious disease by burying themselves into the sub mucosa of the duodenum and feeding on the epithelial cells of the Brunner’s gland resulting in anorexia, profuse fetid diarrhea, drop in plasma protein concentration and anemia, which weaken the host [13]. Mature Paramphistomes are also responsible for rumenitis, irregular rumination, un-thriftiness, lower nutrition conversion and loss of body condition, resulting in considerable economic loss [9].

The species identification is still neglected as the various species of the family Paramphistomatidae are difficult to be detected through a systematic point of view, and most of the reports do not quote the main one [14].

The rumen fluke, *P. cervi* has economic importance in ruminants but presently no reliable methods are available to identify and differentiate this parasite from other Paramphistomes. The present study represents the morphological and histological identification of *P. cervi* using Borax carmine and H and E staining methods, in order to provide a base for future molecular studies.

The present study on morphological and histological identification of *P. cervi* was designed to fulfill the need for molecular understanding of this economically important parasite.

## Materials and Methods

### Collection of adult parasites

The rumen of 350 sheep between 1 and 2 years of age was inspected for the presence of rumen flukes during slaughter at various local abattoirs of Anand, Ahmedabad and Vadodara District in Gujarat state, of which 17 sheep were found positive for Paramphistomes. Infected part of the rumen was brought to the laboratory of Department of Veterinary Parasitology, where it was washed thoroughly. After washing, adult flukes were carefully picked up with the help of forceps from the mucosa of the rumen of naturally infected sheep and then washed 3-4 times with phosphate buffer saline. After that, samples were preserved in 10% buffered formalin until being processed for morphological and histological studies. The samples were carefully labeled with proper details.

### Morphological identification

Rumen flukes were preliminarily identified under microscope using low power magnification and then slides were prepared for detailed morphological studies and identification. The collected flukes were placed on petridish and observed through stereo microscope to appreciate the morphology. Final identification of *Paramphistomum* spp. was done based on morphology of flukes; its shape, anterior sucker, posterior sucker (acetabulum), terminal genitalium and tegumental papillae, following the standard guidelines given by Urquhart *et al*. [15]. Parasites were processed for whole mounting and stained by Borax carmine according to the procedure given by Singh and Srivastava [16].

Out of all recovered adult flukes, 10 flukes were randomly picked from each infected sheep and were washed in water. After washing, flukes were flattened between two glass slides and fixed in Bouin’s fluid at room temperature for 24 h. After that, these specimens were washed in water and then stained for 24 h in 0.5% Borax carmine and subsequently destained in 1% hydrochloric acid until the pink color was observed. The acid was thoroughly washed out from all specimens with water. Thereafter, the specimens were washed with tap water and subsequently dehydrated through 50-100% alcohol for 1 h each, and cleared by xylene for 30 min. The cleared specimens were mounted using DPX and covered with a coverslip. The mounted slides were allowed to the air dry and observed under the light microscope.

### Histological identification

The species thus identified was further confirmed by histological identification of 10 formalin preserved flukes from each sample, which was processed by paraffin embedding method and stained by H and E stain as per Luna [17]. 10 longitudinal and median sagittal sections, each 6-10 µ thick were cut by a Leica RM2125 microtome. The prepared sections were stained with H and E and were examined by microscopy and microphotography in order to identify oral and ventral suckers, testes and ovary. The species were identified according to the criteria outlined by Yamaguti [18], Eduardo [19] and Sey [20].

## Results

It is very difficult to identify and differentiate the species of amphistomes on the basis of its morphology due to significantly less variation in size and shape of parasite and its internal organs, which varies even in mature and immature worms of same spp. Morphology cannot be very well appreciated in the stereo microscope, but can be confirmed well by the histological examination of the parasite.

### Morphological identification

**Figure-1:**
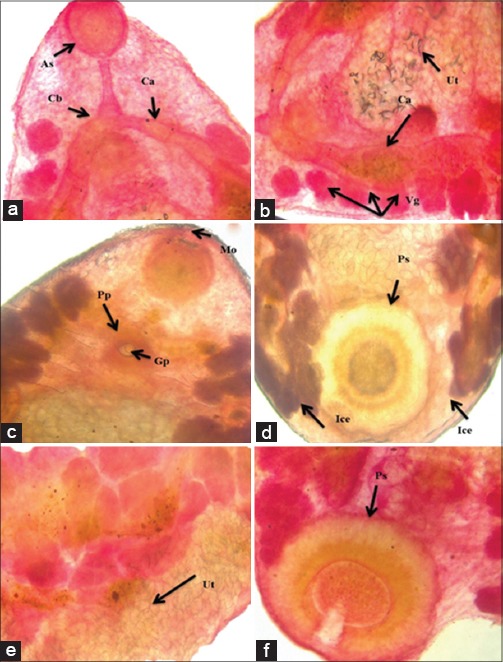
Whole mounted parasite stained with borax carmine showing visceral organs: (a) Anterior sucker (As), cecal bifurcation (Cb), caecum (C), (b) cecum (Ca), vitelline gland (Vg), uterus (Ut), (c) mouth (Mo), pars prostatica (Pp), genital pore (Gp), (d) intestinal cecal end (Ice), (e) uterus (Ut), (f) posterior sucker (Ps).

*P. cervi* is reported in several studies conducted on ruminants [18-20]. Morphological identification of *P. cervi* was carried out on the basis of size and shape of fluke and position of anterior and posterior sucker (acetabulum). In the present study, most of the species were of *P. cervi*, which were found mainly in the rumen and were light pink in color with a sucker at the tip of the cone (Figure-1a) and another sucker ventrally at the posterior end. The body of *P. cervi* was pear-shaped, slightly concave ventrally (conical) and convex dorsally. The worm measures about 3-8 mm in length and 1.5-3.0 mm in width at anterior end of posterior third or at its junction with middle third. Mouth was terminal, funnel-shaped, widened posteriorly. Caeca were wide, pursued a serpentine course and reached anterior level of acetabulum with blind ends more dorsal than lateral (Figure-1d). Genital pore was situated behind intestinal bifurcation (Figure-1c). Acetabulum (posterior sucker) was sub-terminal, about one-fourth to one-fifth of body length (Figure-1f). Clusters of vitelline glands were extended from the pharynx to the posterior sucker and lie between the caeca and the lateral margins of the body (Figure-1b). The uterus was wavy and runs dorsally to testes (Figure-1e).

### Histological identification

The histological identification of *P. cervi* recorded in the present study was done on the basis of histology of flukes; anterior sucker, posterior sucker (acetabulum), terminal genitalium, testes, ovary and on the basis of histological peculiarities of the muscular organs such as pharynx, genital opening and posterior sucker (acetabulum).

**Figure-2:**
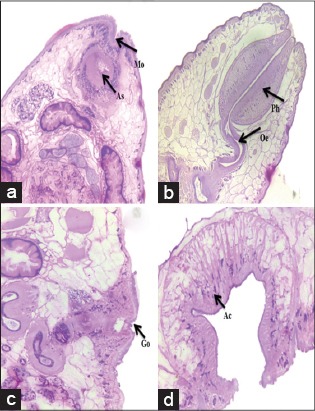
Microscopic pictures showing: (a) Mouth (Mo), anterior sucker (As), (b) *Liorchis* type of pharynx (Ph), (c) *Gracile* type of genital opening (Go) and (d) *Paramphistomum* type of posterior sucker/acetabulum (Ac) in *Paramphistomum* Cervi (H and E, ×150).

**Figure-3:**
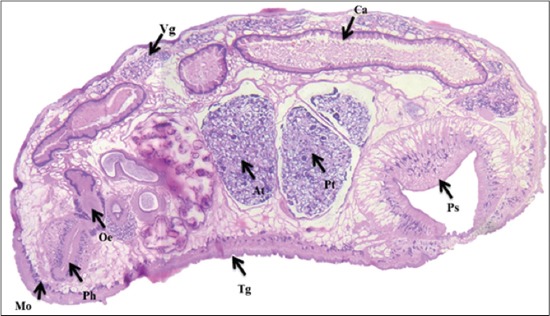
Microscopic picture of medial section of *Paramphistomum cervi* showing different organs: Mouth (Mo), pharynx (Ph), tegument (Tg), caecum (Ca), vitelline gland (Vg), anterior testis (At), posterior testis (Pt), posterior sucker (Ps).

In the present study, it was observed that *P. cervi* had a Liorchis type of pharynx, Gracile type of genital opening and Paramphistomum type of acetabulum in median sagittal section (Figure-2). Cuticle was thick, smooth except at genital pore and anterior extremity, which was covered with numerous prominent papillae. Both anterior and posterior suckers had thick rims covered with transverse folds. Esophagus was bent dorsally; musculature of wall relatively thin, no bulb or the posterior sphincter; lumen lined throughout by hyaline layer (Figure-3).

**Figure-4:**
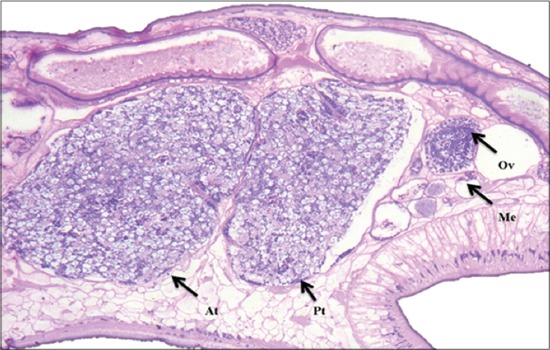
Microscopic picture showing reproductive organs: Distinctly lobed anterior (At) and posterior testis (Pt), ovary (Ov), mehlis gland (Me) (H and E, ×150).

**Figure-5:**
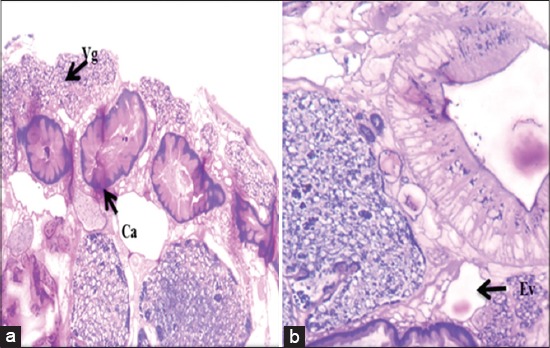
Microscopic picture showing: (a) Caecum (Ca), vitelline gland (Vg), (b) excretory vesicle (Ev) (H and E, ×150).

Testes were distinctly lobed, situated a little obliquely, tandem, in mid-body, pre-ovarian in dorso-ventral direction (Figure-3). Seminal vesicle was long, tubular, strongly coiled, thin-walled and convoluted in front of anterior testis; pars musculosa was very short and weakly developed. Ovary was sub-spherical, un-lobed, post-testicular and pre-acetabular; mehlis gland was close to the ovary; Laurer’s canal crossing excretory vesicle or duct, opening on the dorsal surface (Figure-4). Uterine coils were dorsal to testes and ventral to male ducts. Vitellaria were follicular, lateral, extending from the level of the pharynx to acetabulum, not confluent medially in their posterior or anterior limits. Excretory vesicle was antero-dorsal to acetabulum; excretory pore was anterior to opening of Laurer’s canal at level of posterior testis (Figure-5). The genital pore was situated at the anterior third of the body (behind intestinal bifurcation) with radial muscle fibers. Genital atrium was covered with cuticular papillae which extend over the surrounding body surface. It was encircled by a fold of the body wall (Figure-2).

## Discussion

Morphological and histological identification is important features used to differentiate Paramphistomes. Several earlier studies have investigated morphological and histological variation in *Paramphistomum* species.

The described parasites belong to the genus Paramphistomum [21] (Platyhelminthes: Trematoda: Digenea). The color of fluke, shape and size of body, position of anterior and posterior sucker, presence of genital pore and vitelline glands were similar to the morphological identification system followed earlier by Melaku and Addis [9], Yamaguti [18], Eduardo [19] and Nasmark [22]. Yamaguti [18] collected 20 gravid specimens of which about a dozen were dissected and examined for the terminal genitalia and the ovarian complex of *P. cervi*. Histological identification observed in the present study was similar to the earlier studies carried out by Eduardo [19], Sey [20], Nasmark [22] and Coskun *et al*. [23]. Sey [20] recovered four species of rumen fluke viz. *Paramphistomum cervi*, *P. daubneyi*, *P. gotoi* and *P. ichikawai* from cattle and prepared more than 200 median sagittal sections to study the morphology of *P. cervi* and histomorphology of its muscular organs. He observed that muscular organs of *P. cervi* were of Liorchis (pharynx), Gracile (genital atrium) and Paramphistomum (acetabulum) type, which is similar to this study. Coskun *et al*. [23] collected amphistomes from cattle and compared *P. cervi* from *P. gotoi* on the basis of papillae found in the pharynx and by the position of the blind caeca. Liorchis type of pharynx in *P. cervi* in comparison to calicophoron type of pharynx of *Paramphistomum gracile* [19], Gracile type of genital opening and Paramphistomum type of acetabulum [22] differentiate *P. cervi* from other paramphistomes such as *P. gracile, P. gotoi, Calicophoron calicophorum, Cotylophoron cotylophorum*.

Many scientists studied the Prevalence of various species of amphistomes in sheep in various countries. *P. cervi* was also reported in Nigeria [24,25], Egypt [26], Mexico [27], Thailand [3,28], Iran [29] and Pakistan [30]. *Calicophoron daubneyi* has been reported in Europe, Asia, Africa Oceania and France [31]. *Gastrothylax cruminefer* was found in Nigeria [24], Iran [32] and Kashmir [33]. *Carmyerius synethes* and *Calicophoron microbothrium* were also found in Nigeria from sheep [13]. *P. cervi* was recorded in Kashmir from sheep [34], and in Udaipur of Southern Rajasthan from cattle [35]. In Pakistan *P. cervi* was also reported from buffalo [36] and goats [36,37].

## Conclusion

Morphological and histological studies of all parasites revealed presence of *P. cervi* mainly in adult stage in the rumen of sheep infected with paramphistomosis as the most common species and etiology behind the economic impact of this disease and provides a solid foundation for studying the reproductive biology of Paramphistomes and other related trematodes using molecular techniques. This disease and its impact can be controlled by interrupting the lifecycle of the parasite with prevention of grazing nearby water logged areas and the proper use of effective dewormer.

## Authors’ Contributions

This study is the major component of the work towards the M.V.Sc thesis of Vijayata, under the guidance of JJH. MK and SP helped in histopathology, drafted and thoroughly revised the manuscript. VDC and SP helped in sample collection from various abattoirs. PVP was the member of the advisory committee for the research work and provided guidance. All authors read and approved the final version of the manuscript.
